# Traumatic Porencephaly with Strabismus: A Case Report

**Published:** 2012-07-30

**Authors:** A H Sarmast, H I Showkat, S Farooq Mir, O Masood, A R Kirmani, A R Bhat

**Affiliations:** 1 Department of Surgery, SKIMS, Srinagar, Kashmir, India; 2 Department of Internal Medicine, SKIMS, Srinagar, Kashmir, India; 3 Department of Neurosurgery, SKIMS, Srinagar, Kashmir, India

**Keywords:** Porencephaly, Cyst, Squint, Shunt

Dear Editor,

Porencephalic cyts of the brain are very rare disorders and we present a case of a twelve years old boy who presented with a post-traumatic unilateral strabismus that on imaging proved to be due to a porencephalic cyst.

Porencephaly is a pseudocyst secondary to an infarct or other destructive cerebral lesions.[1] Acquired porencephaly can be caused by various factors such as trauma, infarction, hemorrhage and focal encephalitis destroying cerebral tissue.[2][3][4] Congenital brain lesions include two types of porencephaly; genetic porencephaly, resulting from maldevelopment during early neuronal migration and encephalophaloplastic porencephaly, which is late prenatal or perinatal vascular lesion due to arterial ischemic stroke or venous thrombosis. Porencephalic cysts can be located in any lobe or lobes of the two brain hemispheres,[5] they can be cortical or subcortical, unilateral or bilateral and the location often corresponds to territories supplied by the cerebral arteries.[6]

**Fig. 1:**
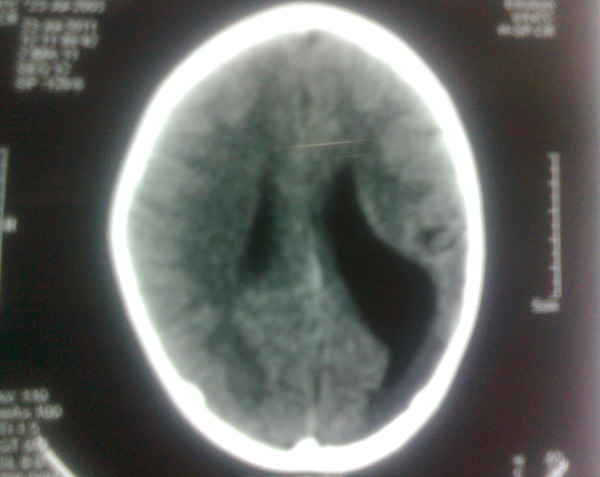
NCCT Head showing porencephaly in the left parieto-occipital region.

A 12-year-old male fell from a height of 10 feet with left side of head hitting the floor. He suffered from loss of consciousness for a few minutes. He was taken to a hospital where his preliminary examination proved to be unremarkable with opthalmological assessment as normal and he was discharged after 6 hours to follow our neurosurgical center in case of any deterioration. For a period of 15 days at home, the patient was completely normal, however on 16th day post-trauma, he complained of diplopia. His parents noticed medial deviation of the left eye. He was brought to our neurosurgical center with these complaints. On admission, he was alert and well oriented. The physical examination was unremarkable except for a paralytic squint of the left eye. Neurological examination showed no deficit. An urgent non-contrast computed tomography (NCCT) scan of the head showed left porencephaly in parieto-occipital region (Figure 1). Bone window was normal. He showed no symptoms or signs of meningoencephalitis. Serial CT scans detected no abnormalities of the brain parenchyma. He was managed with a cystoperitoneal shunt; however his lateral rectus palsy did not recover till six months of post-operative period and was referred to the ophthomological center for further management, where a full width lateral transposition of the vertical rectus muscle combined with a medial rectus recession was done.

Porencephalic cysts vary greatly in size. They are typically cerebrospinal fluid (CSF)-filled cavities with a smooth wall and are lined with gliotic or spongiotic white matter.[6] Antenatal diagnosis of porencephaly by ultrasonography (USG) is possible from the third trimester in case of congenital origin.[7] USG, CT scan and MRI can detect the cyst.[8] The typical porencephalic cyst is a cystic space in the brain parenchyma that communicates with an enlarged adjacent ventricle. The cysts have the same appearance as CSF at all MR sequences. Adjacent white matter typically shows hyperintensity on T2-weighted and FLAIR images.[6] EEG taken over porencephalic cysts are characterized by an increased theta and delta bands in the areas surrounding the lesion sites identified by CT.[9] The differential diagnosis for the porencephalic cyst includes arachnoid cyst, schizencephaly, ependymal cyst, encephalomalacia, and hydranencephaly. Arachnoid cysts are extra-axial and displace the brain cortex away from the adjacent skull. Schizencephaly is a CSF-filled cavity that is lined with heterotopic gray matter and extends all the way from the ventricle to the brain surface. Ependymal cysts are typically intraventricular with normal surrounding brain tissue.[6] Porencephalic cysts are usually associated with various ophthalmic and neurological signs, mainly compressive in origin.[9] It is generally recommended that in the presence of increased intracranial pressure (ICP) or progressive neurological deficits, surgical modes to be adopted to treat these cysts by shunt surgery with or without closure of the defect by a durocranioplasty.[9][10] Placement of a cystoperitoneal or a ventriculoperitoneal shunt in cases of expansive porencephaly with or without hydrocephalus is a form of treatment.[11] Children with intractable seizures and porencephalic cysts benefit from uncapping and cyst fenestration to the lateral ventricle. Concomitant peri-operative complications are mild and are easily treated.[12]
